# Trimethylamine N-Oxide (TMAO) Mediates Increased Inflammation and Colonization of Bladder Epithelial Cells during a Uropathogenic *E. coli* Infection In Vitro

**DOI:** 10.3390/pathogens12040523

**Published:** 2023-03-27

**Authors:** Rongrong Wu, Ashok Kumar Kumawat, Isak Demirel

**Affiliations:** School of Medical Sciences, Örebro University, Campus USÖ, 701 82 Örebro, Sweden

**Keywords:** TMAO, urinary tract infection, UPEC, UTI, inflammation

## Abstract

Urinary tract infections (UTIs) are among the most common infections in humans and are often caused by uropathogenic *E. coli* (UPEC). Trimethylamine N-oxide (TMAO) is a proinflammatory metabolite that has been linked to vascular inflammation, atherosclerosis, and chronic kidney disease. As of today, no studies have investigated the effects of TMAO on infectious diseases like UTIs. The aim of this study was to investigate whether TMAO can aggravate bacterial colonization and the release of inflammatory mediators from bladder epithelial cells during a UPEC infection. We found that TMAO aggravated the release of several key cytokines (IL-1β and IL-6) and chemokines (IL-8, CXCL1 and CXCL6) from bladder epithelial cells during a CFT073 infection. We also found that CFT073 and TMAO mediate increased release of IL-8 from bladder epithelial cells via ERK 1/2 signaling and not bacterial growth. Furthermore, we showed that TMAO enhances UPEC colonization of bladder epithelial cells. The data suggest that TMAO may also play a role in infectious diseases. Our results can be the basis of further research to investigate the link between diet, gut microbiota, and urinary tract infection.

## 1. Introduction

Urinary tract infections (UTIs), often caused by uropathogenic *Escherichia coli* (UPEC), are among the most common infections in humans. Approximately 50–60% of women will experience at least one UTI in their lifetime, and 25–30% of these will have recurrent infections. Hence, UTIs have a great impact on the patient’s quality of life [1]. UPEC has developed a wide range of mechanisms to modulate and evade the innate immune system to successfully colonize the urinary tract. UPEC utilizes virulence factors such as type-1 fimbriae, P-fimbriae, capsule, lipopolysaccharides (LPS), iron acquisition systems, α-hemolysin, SisA, and SisB to cause symptomatic UTIs [1,2]. Studies have shown that UPEC, through type-1 fimbriae, can adhere to, invade, replicate, and form intracellular bacterial communities (IBC) in bladder epithelial cells and that the majority of clinical UPEC isolates have this ability [3,4].

The bladder epithelial cells form an immunoreactive mucosal barrier that protects the urinary tract from infections. During a UPEC infection, host recognition receptors are triggered by different bacterial virulence factors to produce several inflammatory mediators, of which the proinflammatory chemokines and cytokines IL-8 and IL-6 are the most studied [5,6]. Recently, there has also been an increased interest in the role of the proinflammatory cytokine IL-1β during a UTI. IL-1β is one of the most potent inflammatory mediators and has also been implicated in dysregulated inflammation. Some studies propose that IL-1β promotes a protective innate immune response, important for controlling UPEC colonization of the urinary tract [7,8,9]. However, other studies show that IL-1β knockout mice are protected from UTIs [10,11]. This suggests that IL-1β may increase the bladder mucosa’s susceptibility to infection and enhance UPEC colonization.

Trimethylamine N-oxide (TMAO) is a proinflammatory gut microbial-derived metabolite that has been linked to vascular inflammation [8] and atherosclerosis [9]. TMAO is produced through oxidation of trimethylamine (TMA) by flavin monooxygenase 3 (FMO3) in the liver and is eliminated out of the body though urine [12]. TMA is produced from the metabolism of choline, phosphatidylcholine, and L-carnitine by the intestinal flora [12]. The main dietary source of choline, phosphatidylcholine, and L-carnitine are eggs, dairy products, red meat, and fish [13]. High levels of TMAO have been associated with poor prognoses in patients with chronic kidney disease [3], coronary artery disease (CAD) [9], and chronic obstructive pulmonary disease [10]. Furthermore, TMAO is also known to be an alternative exogenous electron acceptor in *E. coli* during both aerobiosis and anaerobiosis [14]. Hence, the role of TMAO in inflammation and bacterial respiration is known. However, the role that TMAO plays in the context of infectious diseases like UTIs remains unclear. The aim of this study was to investigate if TMAO can aggravate bacterial colonization and the release of inflammatory mediators from bladder epithelial cells during a UPEC infection.

## 2. Materials and Methods

### 2.1. Cell Culture

The human bladder epithelial cell line HBLAK (CELLnTEC Advanced Cell Systems AG, Bern, Switzerland) originated from a healthy individual’s bladder and was spontaneously transformed, providing long-term cell growth without senescence. HBLAK cells were cultured in CnT-PR cell culture medium (CELLnTEC) in a humidified atmosphere at 37 °C with 5% CO_2_. HBLAK cells were differentiated for 24 h prior to infection with CnR-PRD (CELLnTEC). The cell culture medium was supplemented with 1 mM CaCl_2_. Gentamicin (50 µg/mL; Sigma-Aldrich, St. Louis, MO, USA) was also added to the cells 24 h prior to infection but was excluded during the experiments, as previously described [15].

### 2.2. Bacteria

The UPEC strain CFT073 was originally isolated from a pyelonephritis patient [16]. The CFT073 strain was maintained on tryptic-soy agar (TSA) (Becton Dickison, Le Pont Claix, France). Prior to the experiments, CFT073 was grown in Difco Luria-Bertani (LB) broth (Lennox; Franklin Lakes, NJ, USA) at 37 °C aerobically on a shaker overnight, as previously described [15].

### 2.3. Stimulation of Bladder Epithelial Cells

The HBLAK cells were pre-incubated with or without TMAO (200 µM, Sigma-Aldrich, St. Louis, MO, USA) for 2 h or an ERK inhibitor PD98059 (10 µM, Santa Cruz Biotechnology Inc., Heidelberg, Germany) for 3 h and then infected with CFT073 (MOI 10) for 24 h at 37 °C in 5% CO_2_. Supernatants were collected and kept at −80 °C until further analysis. A panel of 92 inflammatory proteins were analyzed using proximity extension assay (PEA) technology (Olink Bioscience AB, Uppsala, Sweden). The protein data are presented as normalized protein expression levels (NPX). Proteins below the limit of detection (LOD) were excluded from the analysis, as described previously [17].

### 2.4. Measurement of Cytokine Release and Cell Viability

An enzyme-linked immunosorbent assay (ELISA) was performed to measure IL-1β and IL-8 (ELISA MAX Deluxe Sets, BioLegend, San Diego, CA, USA) in the supernatant from HBLAK cells after infection following the manufacturer’s instructions. The lactate dehydrogenase (LDH) assay (CyQUANT LDH Cytotoxicity Assay Kit, Thermo Fisher Scientific, Waltham, MA, USA) was performed to examine cell viability according to the manufacturer’s instructions. The absorbance signals from both kits were evaluated using the Cytation 3 plate reader (BioTek, Winooski, VT, USA).

### 2.5. Caspase-1 Activity Assay

HBLAK cells were pre-incubated with 50 µM of the caspase-1 substrate Ac-YVAD-AMC (Enzo Life Sciences, New York, NY, USA) for 1 h at 37 °C in the cell incubator with 5% CO_2_. The HBLAK cells were then pre-incubated with TMAO (200 µM) for 2 h prior to CFT073 infection (MOI 10) and then for 3 h at 37 °C in 5% CO_2_. Caspase-1 activity was detected with the Cytation 3 plate reader at 340/440 nm, as previously described [15].

### 2.6. Bacterial Growth Assay

CFT073 (starting with 1 × 0^6^ CFU/mL) was grown in a minimal salt medium (1.3% [wt/vol] Na_2_HPO_4_, 0.3% KH_2_PO_4_, 0.05% NaCl, and 0.1% NH_4_Cl supplemented with 20 mM glucose, 2 mM MgSO_4_, 100 μM CaCl_2_, and 0.25% Casamino Acids) with or without TMAO in a 96-well plate. The 96-well plate was incubated at 37 °C for 24 h, and the bacterial growth was monitored at 600 nm every 10 min with the Cytation 3 plate reader, as previously described [18].

### 2.7. Western Blot Analysis

The bladder epithelial cells were lysed with a RIPA buffer that was supplemented with a protease and phosphatase inhibitor cocktail (Thermo Fisher Scientific, MA, USA). Protein sample concentrations were quantified using the DC protein assay (Bio-Rad Laboratories, Hercules, CA, USA). An amount of 20 µg of protein was mixed with a Laemmli buffer, boiled for 10 min at 95 °C, and separated by 4–15% SDS-polyacrylamide gel electrophoresis and then transferred onto a methanol-activated polyvinylidene fluoride membrane (PVDF) (Bio-Rad Laboratories). The PVDF membranes were blocked with 3% BSA in Tris-buffered saline containing 0.1% Tween 20 (TBST) for 1 h at room temperature, followed by primary antibody incubation overnight at 4 °C. Human phosphorylated ERK 1/2 was detected using a mouse monoclonal antibody (#9106, Cell Signaling Technology, Danvers, MA, USA). Human total ERK 1/2 was detected using a rabbit monoclonal antibody (#4695, Cell Signaling Technology). GAPDH was detected by using a rabbit polyclonal antibody (SC-25788, Santa Cruz Biotechnology, Dallas, TX, USA). As secondary antibodies, mouse anti-rabbit IgG (HRP) (Santa Cruz Biotechnology) and mouse IgGκ light chain-binding protein (m-IgGκ BP, HRP) (Santa Cruz Biotechnology) were used and incubated for 1 h at room temperature. Luminata Forte Western HRP Substrate was used to develop the membranes (Merck Millipore, Darmstadt, Germany), as previously described [15].

### 2.8. Colonization Assay

The HBLAK cells were pre-incubated with TMAO (200 µM) for 24 h prior to CFT073 infection (carrying an eGFP-plasmid, MOI 10) for 4 h at 37 °C in 5% CO_2_. After infection, the cells were washed with PBS, and the adhered/invaded (referred to as colonized) bacteria were quantified and imaged with the Cytation 3 plate reader. Colonization is reported as mean fluorescence intensity (MFI) after subtraction of background fluorescence, as described previously [15].

### 2.9. Data Analysis

All data shown are expressed as mean ± SEM. The differences between the groups were analyzed by one-way ANOVA followed by Bonferroni multiple testing correction or by the unpaired Student’s *t*-test. Statistical significance of the differences was considered at *p* < 0.05.

## 3. Results

### 3.1. TMAO Increases UPEC-Induced Inflammatory Mediator Release

**Figure 1:**
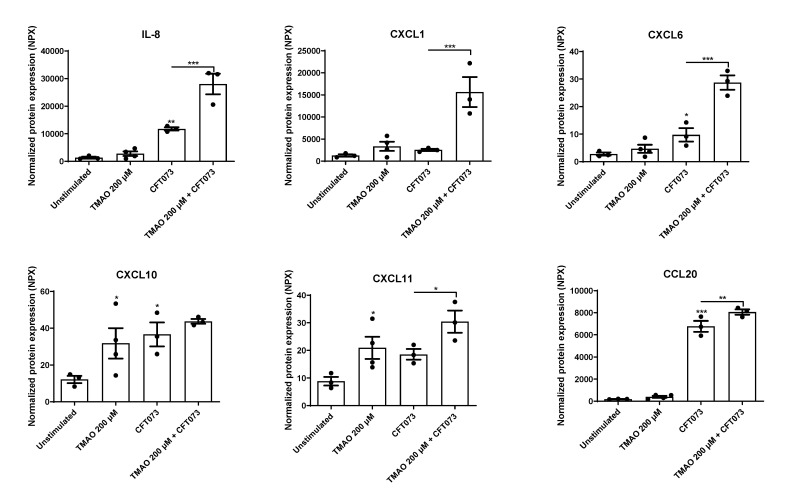
TMAO promotes increased chemokine release. Bladder epithelial cells were pre-stimulated with 200 µM TMAO for 2 h prior to CFT073 (MOI 10) infection for 24 h followed by analysis of protein expression using a panel of inflammation-related proteins. Data are presented as mean ± SEM (n = 3–4 independent experiments). Asterisks denote statistical significance compared to unstimulated cells (* *p* < 0.05, ** *p* < 0.01, *** *p* < 0.001).

The human bladder epithelial cell line HBLAK was pre-stimulated with TMAO for 2 h prior to CFT073 infection (MOI 10) for 24 h, and the chemokine release was evaluated. We found that CFT073 alone significantly increased the release of IL-8, CXCL6, CXCL10, and CCL20 compared to unstimulated cells (Figure 1). We also found that TMAO alone significantly increased the release of CXCL10 and CXCL11 compared to unstimulated cells (Figure 1). The combination treatment with both TMAO and CFT073 had significant additive effects on the release of CXCL11 and CCL20 and synergistic effects on the release of IL-8, CXCL1, and CXCL6 compared to CFT073 infection alone (Figure 1).

**Figure 2:**
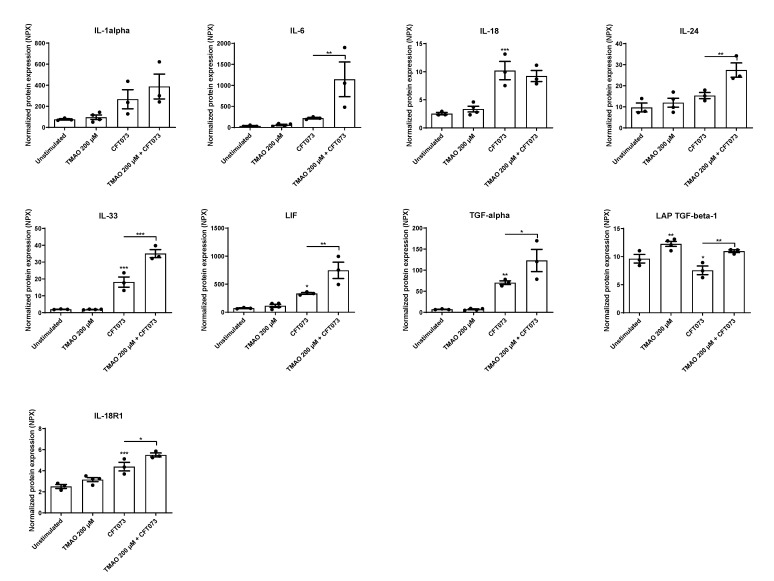
TMAO promotes increased cytokine release. Bladder epithelial cells were pre-stimulated with 200 µM TMAO for 2 h prior to CFT073 (MOI 10) infection for 24 h followed by analysis of protein expression using a panel of inflammation-related proteins. Data are presented as mean ± SEM (n = 3–4 independent experiments). Asterisks denote statistical significance compared to unstimulated cells (* *p* < 0.05, ** *p* < 0.01, *** *p* < 0.001).

We continued to investigate the effects of TMAO on UPEC-mediated cytokine and cytokine receptor release. We found that CFT073 alone significantly increased the release of IL-18, IL-33, LIF, TGF-α, and IL-18R1 compared to unstimulated cells (Figure 2). We also observed that CFT073 significantly reduced and TMAO significantly increased the release of LAP TGF-β1 compared to unstimulated cells (Figure 2). The combination of both TMAO and CFT073 had significant additive effects on the release of IL-18R1 and synergistic effects on the release of IL-6, IL-24, IL-33, LIF, TGF-α, and LAP TGF-β1 compared to CFT073 infection alone (Figure 2).

**Figure 3:**
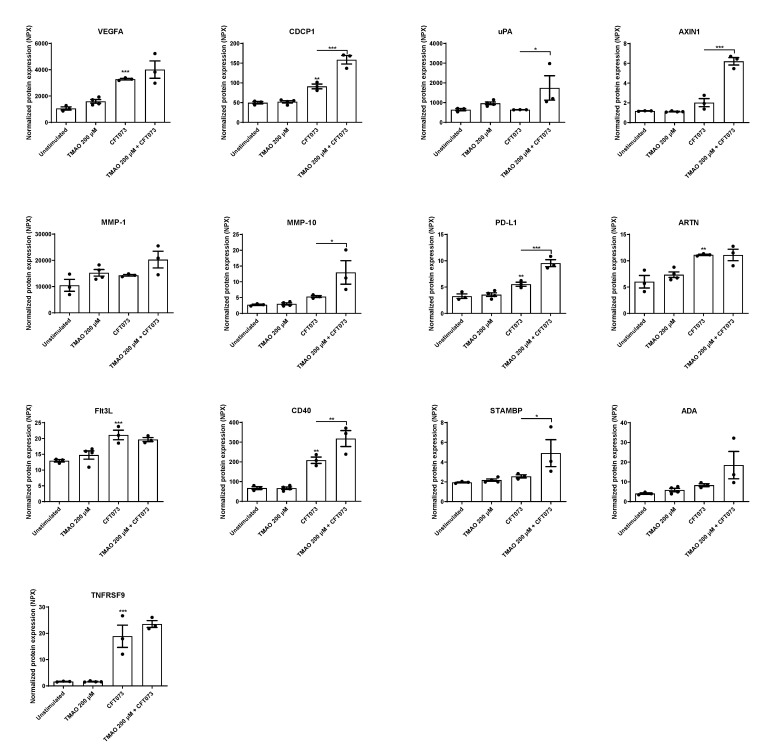
TMAO promotes increased inflammatory mediator release. Bladder epithelial cells were pre-stimulated with 200 µM TMAO for 2 h prior to CFT073 (MOI 10) infection for 24 h followed by analysis of protein expression using a panel of inflammation-related proteins. Data are presented as mean ± SEM (n = 3–4 independent experiments). Asterisks denote statistical significance compared to unstimulated cells (* *p* < 0.05, ** *p* < 0.01, *** *p* < 0.001).

In the next step, we continued to evaluate the release of additional inflammation-related proteins after UPEC and TMAO stimulation. We found that CFT073 alone significantly increased the release of VEGFA, CDCP1, AXIN1, PD-L1, ARTN, Flt3L, TNFRSF9, and CD40 compared to unstimulated cells (Figure 3). The combination treatment with both TMAO and CFT073 had significant additive effects on the release of PD-L1 and STAMBP and synergistic effects on the release of CDCP1, uPA, AXIN1, MMP-10, and CD40 compared to CFT073 infection alone (Figure 3).

### 3.2. TMAO Increases UPEC-Mediated Caspase-1 Activation and IL-1β Release

**Figure 4:**
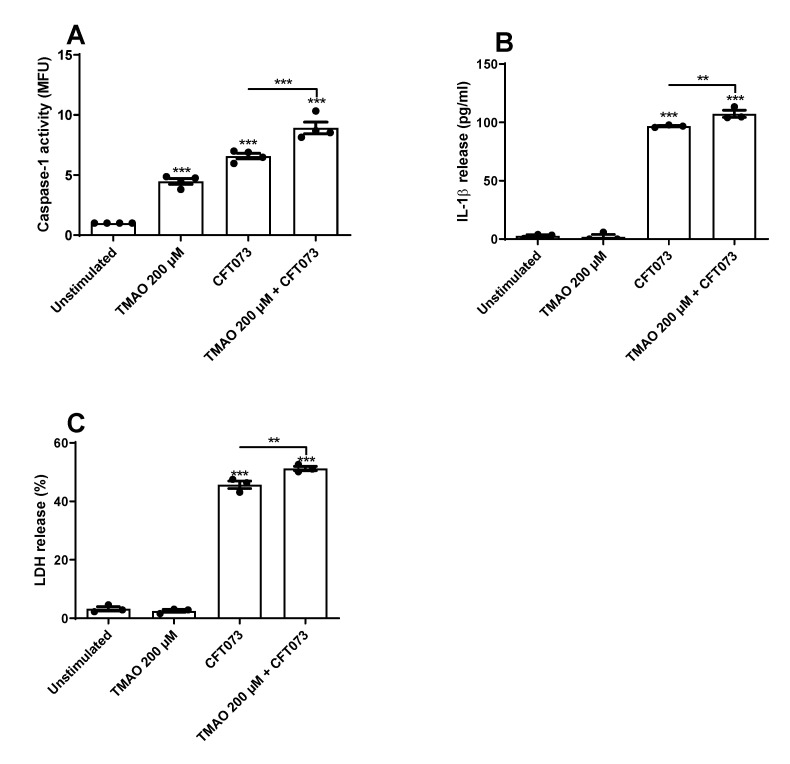
Increased caspase-1 activity and IL-1β release. Bladder epithelial cells were pre-stimulated with 200 µM TMAO for 2 h prior to CFT073 (MOI 10) infection for 3 (**A**) or 6 (**B**,**C**) h followed by analysis of caspase-1 activity (**A**), IL-1β release (**B**), and LDH release (**C**). LDH release is presented as % of total LDH. Caspase-1 activity is presented as fold increase of mean fluorescence units (MFU) compared to unstimulated cells. Data are presented as mean ± SEM (n = 3–4 independent experiments). Asterisks denote statistical significance compared to unstimulated cells (** *p* < 0.01, *** *p* < 0.001).

We continued to investigate if TMAO could increase UPEC-mediated caspase-1 activation and IL-1β release from bladder epithelial cells. We found that both CFT073 and TMAO alone significantly increased caspase-1 activity compared to unstimulated cells (Figure 4A). The combination of TMAO and CFT073 significantly increased caspase-1 activity compared to CFT073 infection alone (Figure 4A). We also found that CFT073, but not TMAO, induced a significant increased release of IL-1β and LDH compared to unstimulated cells (Figure 4B,C). Furthermore, the combination of TMAO and CFT073 significantly increased the release of IL-1β and LDH compared to CFT073 infection alone (Figure 4B,C).

### 3.3. CFT073 and TMAO Mediate the Release of IL-8 from Bladder Epithelial Cells via ERK Signaling

**Figure 5:**
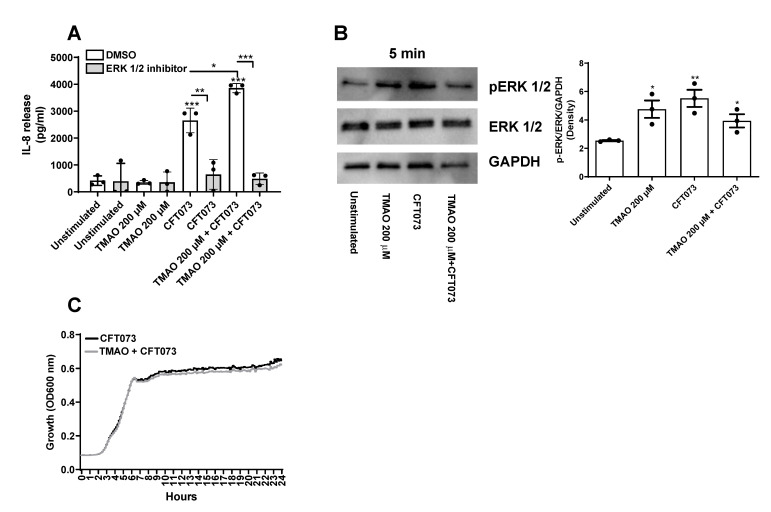
TMAO enhances IL-8 release through ERK1/2. Bladder epithelial cells were pre-incubated with or without TMAO (200 µM) for 2 h or ERK inhibitor PD98059 (10 µM) for 3 h prior to CFT073 infection (MOI 10) for 24 h, followed by evaluating IL-8 release (**A**). Western blot analysis was performed to identify differences in protein levels of p-ERK/ERK after CFT073 (MOI 10) and TMAO (200 µM) stimulation for 5 min (**B**). GAPDH was used as a loading control. Data show CFT073 growth with or without the presence of TMAO (200 µM) for 24 h (**C**). Data are presented as mean ± SEM (n = 3–4 independent experiments). Asterisks show statistical significance compared to unstimulated cells (* *p* < 0.05, ** *p* < 0.01, *** *p* < 0.001).

The next step was to identify the signaling pathway through which TMAO enhances UPEC-mediated inflammation. We chose to use IL-8 as our inflammatory marker, as it is one of the most important chemokines during a UTI [19,20]. We investigated the involvement of ERK1/2, as it has been shown to be important for UPEC-mediated cytokine/chemokine release [15,21,22] and TMAO-mediated inflammation [23,24]. We found, by using a ERK 1/2 inhibitor, that the IL-8 release induced by CFT073 alone or in combination with TMAO was dependent on ERK 1/2 signaling (Figure 5A). In addition, western blot results showed that bladder epithelial cells expressed higher levels of p-ERK1/2 after 5 min of stimulation with CFT073, TMAO, or the combination of TMAO and CFT073 compared to unstimulated cells (Figure 5B). Furthermore, we also investigated if TMAO could enhance bacterial growth. We found that TMAO did not enhance CFT073 growth compared to CFT073 alone (Figure 5C) during 24 h.

### 3.4. TMAO Enhances UPEC Colonization of Bladder Epithelial Cells

**Figure 6:**
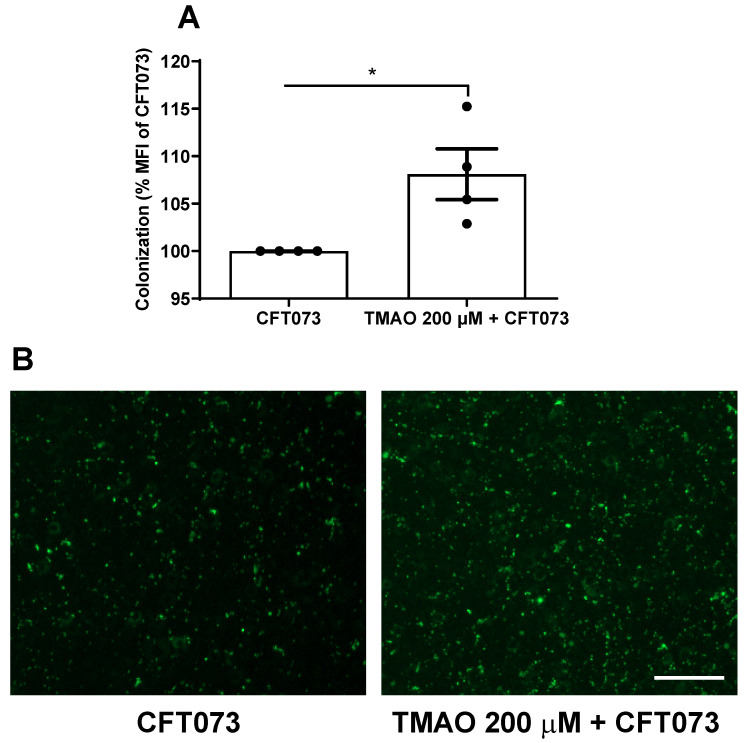
Increased bacterial colonization. Bladder epithelial cells were pre-incubated with TMAO (200 µM) for 24 h prior to CFT073 infection (MOI 10) for 4 h (**A**,**B**). CFT073 (harboring a GFP-expressing plasmid) colonization was quantified as % mean fluorescence intensity (MFI) of CFT073 (**A**) and imaged (**B**). Data are presented as mean ± SEM of n = 4 independent experiments. The asterisk shows statistical significance (* *p* < 0.05). Scale bar: 100 µm.

We proceeded with evaluating the effects of TMAO on UPEC’s ability to colonize (adhere to and invade) human bladder epithelial cells. We found that pre-stimulating bladder epithelial cells with TMAO prior to CFT073 infection mediated a significantly increased bacterial colonization compared to unstimulated cells (Figure 6A,B).

## 4. Discussion

Numerous studies have investigated the role of TMAO in several diseases like chronic kidney disease [3], chronic obstructive pulmonary disease [10], and coronary artery disease (CAD) [9]. However, whether high levels of TMAO can aggravate infectious diseases like UTIs remains unclear. Understanding the interaction between TMAO and bladder epithelial cells during a UPEC infection may help us understand the link between diet, gut microbiota, and urinary tract infections. Our aim was to investigate if TMAO can aggravate bacterial colonization and the release of inflammatory mediators from bladder epithelial cells during a UPEC infection.

The release of inflammation-related proteins from bladder epithelial cells after stimulation with CFT073, TMAO, or the combination of CFT073 and TMAO was evaluated using a multiplex assay that included 92 key proteins. Results obtained from the multiplex analysis revealed that TMAO alone or in combination with CFT073 increases the release of several inflammatory proteins from bladder epithelial cells. Evaluating chemokines, we found that CFT073 induced an increased release of IL-8, CXCL6, CXCL10, and CCL20. TMAO alone increased the release of CXCL10 and CXCL11, and the combination treatment with TMAO and CFT073 increased the release of IL-8, CXCL1, CXCL6, CXCL11, and CCL20 compared to CFT073. IL-8 is an important chemokine vital for the clearance of UPEC [25,26]. Bladder epithelial cells rapidly start to release IL-8 during a UPEC infection, and IL-8 is associated with recruitment of neutrophils to the infection site [27]. Looking at leukocyte migration, we found additional chemokines associated with neutrophil chemotaxis (CXCL1 and CXCL6), lymphocyte chemotaxis (CXCL10, CXCL11, and CCL20), and dendritic cell chemotaxis (CCL20). All these immune cells have recently been associated with the host response during UTIs [22,28,29]. Dendritic cell depletion was associated with delayed neutrophil recruitment and in vivo bacterial clearance [30,31]. The adaptive immune response during a UTI is not as well characterized as the innate immune response. It was shown that mice with B- and T-cell depletion had higher UPEC load post infection compared to wild-type mice [32,33]. Hence, our findings suggest that TMAO alone or in combination with UPEC may contribute to increased influx of neutrophils, lymphocytes, and dendritic cells to the infection site.

Moving on to cytokines, we found that CFT073 increased the release of IL-1β, IL-18, IL-33, LIF, TGF-α, and IL-18R1, but TMAO alone only increased the release of LAP TGF-β1. The combination of TMAO and CFT073 increased the release of IL-1β, IL-6, IL-24, IL-33, LIF, TGF-α, and IL-18R1 compared to CFT073 alone. IL-6 is rapidly release by the bladder epithelium upon a UPEC infection. IL-6 deficient mice exhibited increased UPEC load, reduced antimicrobial peptide release, and increased mortality [34]. IL-1β, IL-18, and IL-33 are members of the IL-1 superfamily, and IL-1β has been linked to the host response during a UPEC-mediated UTI. However, this link is contradictory. Some propose that IL-1β protects the urinary tract from UPEC [7,8,9], and others suggest that IL-1β may enhance UPEC colonization of the urinary tract [10,11]. IL-18, IL-18R1, IL-33 LIF, IL-24, TGF-α, and LAP TGF-β1 at present lack a known role during a UTI. However, they are involved in other inflammatory diseases [35]. We also found that TMAO could significantly increase the activity of caspase-1 alone and in combination with CFT073, which explains the increased IL-1β release. However, TMAO did not induce any cell death, which might explain why TMAO alone does not mediate a release of IL-1β, as IL-1β release has been associated with pyroptosis in bladder epithelial cells [15,36]. The Olink multiplex evaluation also showed that VEGFA, CDCP1, AXIN1, PD-L1, ARTN, Flt3L, CD40, uPA, MMP-10, and STAMBP were altered by CFT073 or TMAO, but they currently lack a known link to the immune response during a UTI. Taken together, TMAO seems to enhance the release of some key cytokines during a UPEC infection, which might affect UPEC’s ability to colonize the urinary tract.

Next, we proceeded with evaluating how TMAO mediates the increased release of inflammatory mediators from bladder epithelial cells. We started by investigating the signaling pathway involved in IL-8 secretion as a model. We found that ERK 1/2 was associated with the release of IL-8 upon CFT073 and TMAO stimulation. We then showed that both CFT073 and TMAO could respectively increase the phosphorylation of ERK 1/2 in bladder epithelial cells. We and others have shown that ERK 1/2 is important for UPEC-mediated cytokine/chemokine release [15,21,22] and TMAO-mediated inflammation [23,24]. Furthermore, we also evaluated if TMAO by itself could increase the bacterial growth of CFT073 and through that be responsible for the increased release of inflammatory mediator. However, we found that TMAO did not induce increased CFT073 growth. TMAO is recognized to be an alternative exogenous electron acceptor in *E. coli* during anaerobic and aerobic conditions [14], but in our setup, it seems that TMAO may not have been needed to promote UPEC growth. Taken together, our findings indicate that ERK 1/2, at least partly, mediates the effect of TMAO on the release of inflammatory mediators, such as IL-8, from bladder epithelial cells.

UPEC’s ability to adhere to and invade bladder epithelial cells is crucial for the colonization of the urinary tract [3,37]. We found that bladder epithelial cells stimulated with TMAO mediated an increased CFT073 colonization. Intracellular UPEC can replicate and form biofilm-like communities and eventually efflux out from their intracellular niche into the bladder lumen to colonize and invade neighbouring bladder epithelial cells [3,37]. Our results suggest that TMAO may modulate bladder epithelial cells, making them more susceptible to UPEC colonization.

In conclusion, we showed that TMAO affects bladder epithelial cells by aggravating bladder inflammation and promoting UPEC colonization. The TMAO-induced increase of IL-8 was mediated by ERK 1/2 and not by bacterial growth. These findings can be the basis of further research to investigate the link between diet, gut microbiota, and urinary tract infections. The increased inflammation induced by TMAO can be positive or negative in terms of clearing the infection. This, in combination with the observed increased colonization of bladder epithelial cells, emphasizes the need to evaluate TMAO effects on UTI in an in vivo mouse model. The in vivo experiments would help clarify if TMAO mediates increased colonization and dysregulated inflammation or if the inflammatory response would help clear the UPEC infection.

## Data Availability

The data presented in this study are available on request from the corresponding author. Share upon request.
